# Extracellular vesicles: A new paradigm in understanding, diagnosing and treating neurodegenerative disease

**DOI:** 10.3389/fnagi.2022.967231

**Published:** 2022-11-03

**Authors:** Ghulam Hassan Dar, Raied Badierah, Erica G. Nathan, Mohmad Abass Bhat, Abid Hamid Dar, Elrashdy M. Redwan

**Affiliations:** ^1^Department of Biochemistry, S.P. College, Cluster University Srinagar, Srinagar, India; ^2^Hassan Khoyihami Memorial Degree College, Bandipora, India; ^3^Department of Biological Sciences, Faculty of Science, King Abdulaziz University, Jeddah, Saudi Arabia; ^4^Medical Laboratory, King Abdulaziz University Hospital, King Abdulaziz University, Jeddah, Saudi Arabia; ^5^Department of Oncology, Cambridge Cancer Center, Cambridge, United Kingdom; ^6^Department of Biotechnology, School of Life Sciences, Central University of Kashmir, Ganderbal, India; ^7^Protein Research Department, Genetic Engineering and Biotechnology Research Institute (GEBRI), The City of Scientific Research and Technological Applications (SRTA-City), Alexandria, Egypt

**Keywords:** extracellular vesicles, neurodegenerative diseases, biomarkers, heterogeneity, drug delivery

## Abstract

Neurodegenerative disorders (NDs) are becoming one of the leading causes of disability and death across the globe due to lack of timely preventions and treatments. Concurrently, intensive research efforts are being carried out to understand the etiology of these age-dependent disorders. Extracellular vesicles (EVs)—biological nanoparticles released by cells—are gaining tremendous attention in understanding their role in pathogenesis and progression of NDs. EVs have been found to transmit pathogenic proteins of NDs between neurons. Moreover, the ability of EVs to exquisitely surmount natural biological barriers, including blood-brain barrier and *in vivo* safety has generated interest in exploring them as potential biomarkers and function as natural delivery vehicles of drugs to the central nervous system. However, limited knowledge of EV biogenesis, their heterogeneity and lack of adequate isolation and analysis tools have hampered their therapeutic potential. In this review, we cover the recent advances in understanding the role of EVs in neurodegeneration and address their role as biomarkers and delivery vehicles to the brain.

## Introduction

Neurodegenerative disorders (NDs) such as Alzheimer’s, Huntington’s, Parkinson’s and amyotrophic lateral sclerosis (ALS) are heterogeneous diseases that follow a progressive pattern of accumulation and spreading of misfolded proteins whose composition varies according to the disease. Accumulation of misfolded proteins results in progressive loss of specific neurons and synapses in distinct regions of the brain (Budnik et al., 2016; Gan et al., 2018). At present, NDs are the second leading cause of deaths worldwide that cannot be cured or slowed down significantly due to lack of treatments. Death of the neurons at different regions of the brain results in a progressive dysfunction of motor control, mood disorders and cognitive impairment that eventually progress to a full-fledged dementia, causing devastating effects on socio and economic life of patients and their families (Dugger and Dickson, 2017). NDs are becoming a global public health challenge due to an increase in the number of deaths and disabilities caused by these diseases. It is estimated that around 50 million people are suffering from NDs and its number is anticipated to increase to 135 million by 2050 (Livingston et al., 2020). To tackle the growing challenges of the neurodegenerative diseases, comprehensive and collaborative efforts need to be developed for effective treatments that could delay onset or retard progress of these diseases.

Over the last few decades, advancement in human genetic studies and development of advanced molecular biology techniques have revolutionized our understanding in determining the causes of various NDs (Paulson, 2009; Alves et al., 2018; Caron et al., 2018). In early 1990s, mutations underlying the common monogenic neurological disorders such as Huntington’s disease, Duchenne muscular dystrophy and Charcot-Marie-Tooth disease type 1 were identified and characterized. Recent advances in DNA sequencing has accelerated the discovery of highly penetrant single-gene mutations of various rare disorders (Ravi et al., 2021). Using advanced genetic approaches, it is now possible to detect and manage the symptoms of the disease before its onset. More importantly, the monogenic nature of several NDs have led to development of therapeutic intervention to either target the mutant genes using antisense RNA and RNA interferences or target the mutant protein by designing chemical inhibitors and monoclonal antibodies (Gabathuler, 2010; Alyautdin et al., 2014; Sharma et al., 2019).

Apart from genetic studies, compelling evidence from cellular, biochemical, and animal studies have shown that misfolding of proteins, their aggregation and accumulation are one of the main causes for the death of neurons, loss of synaptic connections that ultimately leads to brain damage (Soto and Pritzkow, 2018). Despite the fact that the distinctive proteins are involved in various NDs, the process of protein misfolding, their intermediates and end-products are mostly similar, which has provided in-depth understanding about overlapping disease mechanisms in patients with sporadic and inherited neurodegenerative disease (Soto, 2003; Gandhi et al., 2019; Mukherjee et al., 2021). Moreover, high resolution structural studies of protein aggregates revealed presence of conformational variant of the protein aggregates of prion proteins, tau proteins, α-synuclein and β-amyloids that provides molecular basis for presence of distinct tauopathies of each misfolded proteins (Hauw et al., 2015; Lazaro et al., 2019; Lim, 2019).

## Overview of extracellular vesicles

Despite extensive knowledge gained in understanding the molecular mechanism of the aggregation of misfolded proteins and their cross-seeding with other protein aggregates, very little is known about the cellular pathways implicated in spreading of misfolded seeds across different neurons (Soto and Pritzkow, 2018; Liu et al., 2021). Among several cellular pathways proposed, the role of extracellular vesicles (EVs) is gaining extensive importance during the past several years. EVs represent a new paradigm of cell-cell communication that has provided in-depth knowledge in understanding the bidirectional communication between cells and their complex microenvironment. Originally, exosomes were first described by E G Trams in 1981 who observed release of small vesicles of around 40 nm into cell culture media. These vesicles referred to as “exosomes” were found encapsulated within a larger vesicle of 5–10 μm in diameter (Trams et al., 1981). In 1983, Harding and colleagues, while working on trafficking of transferrin receptors, discovered fusion of large multivesicular bodies (MVB), containing small intraluminal vesicles, with the plasma membrane, releasing these small intraluminal vesicles into the extracellular environment. In 1987 Johnstone named these vesicles as exosomes (Johnstone et al., 1987). However, till 1990 exosomes were considered as garbage cans used by cells to discard unwanted cellular proteins. Subsequent study in 1996 by Raposo et al. (1996) which observed activation of CD4^+^ T cells by MHC II enriched exosomes, sparked a renewed interest in exosome biology that led to exponential development of the EV field. Over the past two decades, there have been ground breaking reports demonstrating the role of EVs in cancer metastasis, diagnostics, progression of neurological diseases and application of bioengineered EVs in drug delivery systems (El Andaloussi et al., 2013; Murphy et al., 2019). Meanwhile, Significant progress has been made in understanding the cell biology of exosome biogenesis and their content (Kang et al., 2021). Depending on cells of origin, mechanism of biogenesis, environmental conditions, epigenetic changes and developmental stages, the molecular constituents of EVs include DNA (ssDNA, dsDNA, mtDNA) RNA (miRNA, lncRNA, tRNA, snoRNA), lipids and specific proteins (ESCRT complexes, ALIX, or Rab GTPases, GAPDH, CD9, CD63, CD81, HSP70, and HSP90, tubulin) (Jurj et al., 2020). With the immense interest in EVs and advances in the high throughput techniques, there has been a data explosion of EVs during the last decade. To get an access of the publicly available datasets, an online compendium of heterogeneous databases have been curated by biologists and uploaded into different databases such as Exocarta (Keerthikumar et al., 2016), Vesiclepedia (Kalra et al., 2012), and EVpedia (Kim et al., 2015). Exocarta is mostly focussed on exosome markers and contains information on isolation and characterization methods of these vesicles. Vesiclepedia and EVpedia catalog data from different types of EVs.

Based on the underlying biogenesis and biophysical characteristics, EVs have been categorized into several classes but small EVs with diameter of 30–150 nm are the most well-studied (Table 1; Tkach and Thery, 2016; Willms et al., 2018; Kalluri and LeBleu, 2020). The term exosomes is often referred to vesicles of 30–150 nm in diameter that are generated by inward budding of late-endosomes producing intraluminal vesicles (exosomes) within MVB. The MVBs fuse with the plasma membrane, releasing exosomes into extracellular space (Escola et al., 1998; Stuffers et al., 2009; Raposo and Stoorvogel, 2013; Bebelman et al., 2018; van Niel et al., 2018). Another type of vesicles referred to as microvesicles or ectosomes or microparticles are secreted by cells *via outward* blebbing of plasma membrane. Microvesicles have a characteristic size of 100–1,000 nm (Ostergaard et al., 2012; Raposo and Stoorvogel, 2013; Zaborowski et al., 2015). A third, less studied category of vesicles, known as apoptotic bodies (1–5 μm) are released by blebbing of dying cells (Stahl et al., 2019). Although the above classification system has enabled us to differentiate different types of EVs based on size and other limited biochemical properties, linking size of EVs with their biological functions remains enigmatic (Tkach et al., 2018; Margolis and Sadovsky, 2019). Due to inadequate EV separation and analysis techniques, it is difficult to interpret whether a single cell produces EVs of different sizes or does different sizes of EVs are released by different cells? Similarly, no information is available to understand variation in secretion of EV types at different times and conditions. Due to complexity of EV biology, we are unable to link different types of EVs with their characteristic biological functions. In order to address EV heterogeneity, different new tools and techniques are being applied to characterize several types of EVs. By employing asymmetric-flow field-flow fractionation (AF4), Zhang H. et al. (2018) were able to efficiently resolve EV subpopulations and identified a distinct abundant population of non-membranous nanoparticles named “Exomere” (∼35 nm), which were enriched with proteins involved in metabolism, coagulation and hypoxia. They were also able to separate and characterize two discernible exosome subpopulations, small exosomes vesicles (Exo-S, 60–80 nm) and large exosome vesicles (Exo-L, 90–120 nm). Exo-S were predominantly enriched in protein associated with endosomes, MVB and phagocytosis while as Exo-L were found to contain protein of plasma membrane, cell-cell contact and late-endosomes suggesting that Exo-S are more likely canonical exosomes derived from endosomes and Exo-L are more likely microvesicles that are blebbed from plasma membrane. Recently, studies carried out by Zhang et al. (2021) have identified another amembranous nanoparticle termed as supermere (supernatant of exomere). Although, supermere are morphologically identical to exomere, their protein and RNA content differs from sEVs and exomere. Several clinically relevant proteins such as amyloid precursor protein (APP), cellular-mesenchymal-epithelial transition factor (MET), argonaute-2 (AGO2), TGFβ-induced (TGFBI), and extracellular RNA previously reported to be in exosomes, were highly enriched in supermeres (Zhang et al., 2021).

**TABLE 1:** Classification of Extracellular vesicles and their characteristics.

Name of the vesicles	Type	Protein markers	Size (nm)	References
Small exosomes	Exosomes	Flotillin 1, flotillin 2, Tetraspanin (CD63, CD81, CD9) ESCRT-1	60–80	Thery et al., 2001; Morita et al., 2007; Zhang S. et al., 2018; Doyle and Wang, 2019
Large-exosomes	Exosomes	Annexin A1/A4/A5, charged multivesicular body protein 1A/2A/4B/5 (Hsp40)	90–120	Zhang S. et al., 2018; Jeppesen et al., 2019
Microvesicles	Microvesicles	CD40, selectins, integrins, and cytoskeletal proteins	150–1,000	Heijnen et al., 1999; Di Vizio et al., 2012; Zhang S. et al., 2018
Exomere	Nanoparticle	HSP90AB1, ACTG1, RAB7A, Hist1h3b	35	Zhang S. et al., 2018
Apoptotic bodies	Apoptotic EVs	Annexin V (PS exposure)	>1,000	Thery et al., 2001; Ratajczak and Ratajczak, 2020

To identify and characterize different subpopulation of EVs and other biological nanoparticles, various advanced techniques such as high-resolution density gradient fractionation, immunoaffinity, imaging flow cytometry (IFCM) were recently employed (Gorgens et al., 2019; Jeppesen et al., 2019; Dar et al., 2021). However, ambiguity in biochemical markers associated with EV subtypes and lack of standardized method to isolate different types of EVs across biological samples have remained one of the major bottlenecks in addressing heterogeneity and classification of EVs. Keeping these issues in focus, we prefer to use the latest guidelines from the International Society for Extracellular Vesicles (ISEV) clearly on definition, isolation and classification of EVs. While going through the literature of EVs pertaining to their role in neurodegeneration, we found that many authors have used the term exosomes while describing their results. No specific exosomal marker or method of selectively isolating exosomes were mentioned during characterization of EVs. Therefore, the mentioned exosomes in many cases could also contain small amounts of other types of EVs. In this review, the generic term EVs have been used to mention either exosomes or microvesicles without specific attention to their size, biogenesis and method of isolation. Wherever exosome or microvesicles term have been used, it signifies the choice of the authors in the respective work cited in this review.

## Role of extracellular vesicles in dissemination of pathogenic protein aggregates in neurodegenerative disorders

Despite the findings that showed important physiological roles of central nervous system (CNS) derived EVs in regulation of inflammatory responses (Bianco et al., 2005), promotion of neurite outgrowth (Wang et al., 2011) and regulation of myelin membrane biogenesis (Bakhti et al., 2011), EVs are considered to contribute to the pathogenesis of many neurological diseases, including NDs *via* disseminating pathogenic protein aggregates (Table 2) (Soria et al., 2017; DeLeo and Ikezu, 2018). Although the exact mechanism involving the role of EVs in spreading of aggregated proteins is still unknown, elucidating the pathways and mechanisms by which an abnormal isoform of the host encoded prion protein, referred to as PrP^Sc^, spreads through host have highlighted direct involvement of EVs in spreading of infected prion proteins. Studies carried out by Vella et al. (2008) have demonstrated that both normal (PrP^C^) and abnormal (PrP^Sc^) prion proteins are associated with exosomes secreted by both neuronal and non-neuronal culture cells. By using sucrose density gradient and electron microscopy experiments, the author has found exclusive association of prion proteins with exosomes (Vella et al., 2008). Earlier, it has been demonstrated that endocytic pathways play an important role in conversion of PrP^c^ to PrP^sc^, suggesting the role of endosome-derived exosomes in secretion of PrP^sc^ (Borchelt et al., 1992). Using specific exosome markers, MS and morphological analysis, both PrP^c^ and PrP^sc^ have been found to be released *via* endosome derived exosomes (Fevrier et al., 2004). However, prion association has also been found associated with microvesicles (Caughey et al., 2009; Heumuller et al., 2022; Kovac and Curin Serbec, 2022). Studies carried out by Leblanc et al. (2006) have found that cells infected with retrovirus strongly enhance the release of PrP^C^ and PrP^Sc^ proteins *via* exosomes. Exosomes have been also reported in transmitting infectivity between heterologous cell types, suggesting their role in transmission of prion infectivity within the nervous system (Leblanc et al., 2006; Vella et al., 2007). Inhibition of neutral sphingomyelinase by GW4869 was shown to impede both exosomes biogenesis and packaging of prion proteins, suggesting the important role of exosomes in secretion of prion proteins.

**TABLE 2:** Current knowledge about role of EVs in Pathophysiology of NDs.

Disease type	Key role of EVs	Key markers found in EVs
Alzheimer’s disease	EVs isolated from AD patients transferred toxic Aβ to neurons with subsequent toxic effect on the recipient cells (Falker et al., 2016; Sardar Sinha et al., 2018). Inhibition of EV significantly reduced tau propagation *in vitro* and *in vivo* (Asai et al., 2015). EVs from AD patients induced tau pathology in mice (Winston et al., 2016).	Presence of Aβ and p-tau in brain-derived EVs isolated from blood of patients (Saman et al., 2012; Simic et al., 2016) Upregulation of miR-9-5p, miR342-3p and miR-598 in the blood and CSF of AD patients (Manna et al., 2020; Wang and Zhang, 2020). Elevated levels of autolysosomal proteins (LAMP-1 and cathepsin D) in nEV (Goetzl et al., 2015b).
Parkinson’s disease	Pathogenic species of α-synuclein were found in EVs isolated from CSF of PD patients that trigger oligomerization of soluble α-synuclein in targeted cells (Danzer et al., 2012; Stuendl et al., 2016). EVs containing oligomeric α-synuclein spread Synucleinopathy in mice brain after focal administration (Zhang S. et al., 2018).	Elevated levels of α-synuclein were detected in CSF-derived EVs from PD patients at early stages of the disease (Stuendl et al., 2016). Levels of α-synuclein in EVs of dementia patients with Lewy bodies correlate with severity of the disease Altered miRNA levels in CSF EVs of PD patients (Gui et al., 2015).
Amyotrophic lateral sclerosis	Misfolded superoxide dismutase (SOD 1) transmission between cells occurs *via* EVs (Grad et al., 2014). EVs containing mutant TDP-43 caused degeneration of neurons *in vitro* (Sproviero et al., 2018).	Expression of TDP-43 full-length and C-terminus fragments were reported in a cohort of ALS-FTD patient-derived EVs of CSF origin (Ding et al., 2015).
Huntington’s disease	EVs were found involved in propagation of mHTT in mouse models (Jeon et al., 2016; Ananbeh et al., 2021).	None

Since most of the NDs share common mechanism of aggregation and propagation of misfolded protein to defined regions of the brain, evidence is mounting that EVs carry prion-like templating protein aggregates of other neurodegenerative diseases including Parkinson’s disease (PD), Alzheimer’s disease (AD), Huntington’s disease (HD), and ALS (McAlary et al., 2019; Sandau et al., 2020; Yoon et al., 2022). Once these EVs are secreted by the affected neurons, they are taken up by other cells of the CNS, resulting in transferring of these seed-proteins into the targeted cells. In AD, deposits of amyloid-β (Aβ) proteins and hyper-phosphorylated tau protein aggregates known as neurofibrillary tangles (NFTs) are thought to act as “seeds” that undergo nucleated polymerization leading to formation of pathogenic aggregates in brains of Alzheimer patients (Nizynski et al., 2017; Sarnataro, 2018). APP is a ubiquitous type-1 transmembrane protein consisting of a single transmembrane domain with large extracellular domain and a short cytoplasmic C-terminus domain. Under physiological conditions, cleavage of APP protein on surface of plasma membrane by α-secretase and then γ-secretase results in the formation non-amyloidogenic fragments along with soluble amyloid precursor protein fragments α (sAPPα) and C-terminal fragments (CTFs). Under certain conditions, the extracellular cleavage is made more distant from the membrane by β-site APP cleaving enzyme (BACE) or β-secretase and γ-secretase, leading to formation of a somewhat longer peptide fragment (40–42 AA) called amyloid-beta or Aβ peptide, along with soluble amyloid precursor protein fragments β (sAPPβ) and CTFs (Ehehalt et al., 2003; O’Brien and Wong, 2011; Miura et al., 2020). APP can be processed on the cell surface by α and γ-secretase or reinternalized in clathrin-coated pits into an endosomal compartment containing the proteases BACE1 and γ-secretase. The latter results in the production of Aβ that can be either degraded in the lysosomes or dumped into extracellular space. FRET and other experimental evidence have indicated that BACE interacts with APP in the endosomes and preventing of surface endocytosis blocks almost 80% of Aβ release (Koo and Squazzo, 1994; Kinoshita et al., 2003; Fukumori et al., 2006; Zou et al., 2022). These experimental evidence indicates the role of exosomes in releasing Aβ peptides into the extracellular spaces and their uptake by other neuronal cells. Direct evidence for the involvement of exosomes in neuron-neuron transfer of toxic proteins comes from Sardar Sinha et al. (2018) studies who demonstrated that intracellular levels of lower molecular weight Aβ oligomers (oAβ) and protofilaments is increased in the exosomes isolated from Alzheimer’s patients brain when compared to similar preparations of control brain samples from patients deceased from non-neurological reasons. While studying the release of (Aβ) peptides into the extracellular space, Rajendran et al. (2006) have shown β-secretase cleavage occurs in early endosomes. A fraction of Aβ peptide is trafficked into MVB for extracellular release *via* exosomes. Moreover, exosome markers such as Alix and flotillins have been found in the plaques of AD patients. The staining pattern was absent in the postmortem samples of healthy individuals, suggesting specific association of EVs with amyloid plaques (Saman et al., 2012; Goetzl et al., 2015a; Hu et al., 2016). Numerous other studies indicate that EVs play a prominent role in pathogenesis of AD (Vella et al., 2016; Deng et al., 2017). Studies carried out on neuroblastoma P*^r^*P^C^ knockout cell lines revealed preferential binding and fibrillization of small Aβ species on exosomes expressing P*^r^*P^C^ (Falker et al., 2016; Sardar Sinha et al., 2018). In another study, microglia-derived microvesicles were found to induce Aβ neurotoxicity *in vitro* by promoting formation of small soluble neurotoxic species from Aβ extracellular aggregates (Joshi et al., 2014). These observations reinforces the role of EVs in pathogenesis of NDs in a prion-like manner, thus, making them an alternative pharmaceutical target to downregulate their formation or secretion by inhibiting proteins involved in biogenesis of exosomes (Bulloj et al., 2010; Dinkins et al., 2014; Vella et al., 2016). However, contrary to these results, Yuyama et al. (2014) demonstrated that infusion of neuronal exosomes into brain of APP transgenic mouse decreased Aβ and amyloid depositions, possibly *via* binding of Aβ peptide on neuronal derived exomes, containing abundant glycosphingolipids. These Aβ bound exosomes were believed to be phagocytosed by microglial cells for degradation of Aβ aggregates (Yuyama et al., 2014). It has been found that during excessive formation of Aβ aggregates, microglial cells are unable to degrade all Aβ aggregates and thereby release soluble Aβ peptides *via* microvesicles that induce toxicity to surrounding neurons (Gouwens et al., 2018). Microglia cells have also been demonstrated to play an important role in propagation of tau protein and neurotoxicity in the brain. Using two different tau mouse models, it has been suggested that microglia cells phagocytose tau-containing cytopathic neurons (known as phagoptosis) or tau-containing inactive synapses, which is known as synaptic pruning and secrete tau protein in exosomes, which efficiently transmit tau to neurons (Schafer et al., 2012; Brown and Neher, 2014; Asai et al., 2015). Moreover, the microglial depletion significantly reduces exosomal transmission of tau to neurons *ex vivo*. Similarly, exosomes isolated from the plasma of AD patients were found to induce the formation of tau pathology in CNS of normal mice (Winston et al., 2016). These and other clinical studies strongly support the findings that exosomes are an important component of the pathobiology of Alzheimer’s disease. However, not all aggregates are encapsulated in EVs as majority of extracellular aggregates of tau and α-synuclein are found membrane free and use other cellular pathways for propagation (Mao et al., 2016).

EVS have also been shown to play an important role in spreading of ALS, also known as Lou Gehrig’s disease, after the baseball player who was diagnosed with it (Ferrara et al., 2018; Gagliardi et al., 2021). ALS is a progressive neurodegenerative disease that causes deterioration of motor neurons that control voluntary muscle movement such as walking, eating, breathing, and speaking. Like other NDs, histology of ALS patients revealed abnormal accumulations of protein aggregates in motor neurons and neuronal accessory cells (Ilieva et al., 2009; Sproviero et al., 2018). The major protein aggregates found in ALS include TAR DNA-binding protein 43 (TDP-43) Cu/Zn superoxide dismutase 1 (SOD1), and fused in sarcoma (FUS). Growing evidence suggests that misfolding of these proteins accumulates in the cells and can cause aggregation of their native counterparts through a mechanism similar to self-seeding of infectious prion proteins (Polymenidou and Cleveland, 2011). In ALS it is observed that the release and propagation of protein aggregates of SOD 1, TDP-43, and FUS occurs *via* EVs (Sproviero et al., 2018; You and Ikezu, 2019). Astrocytes overexpressing mutant SOD 1 were found to secrete a greater number of exosomes and efficiently transfer mutant SOD 1 to spinal neurons, leading to death of these neurons (Basso et al., 2013). Similarly, exposure of N2a cells to EVs isolated from the brains of ALS patients caused redistribution of TDP-43 aggregates in the cells while no such effect was found when EVs isolated from a control brain were added to these cells (Feneberg et al., 2014).

Parkinson’s disease, which is the second most prevalent neurodegenerative disorder, is characterized by intracellular accumulation of protein aggregates composed primarily misfolded and fibrillary forms of α-synuclein, forming intracytoplasmic inclusion bodies known as Lewy bodies at many region in the brain including substantia nigra, locus coeruleus, cerebral cortex, central and peripheral divisions of the autonomic nervous system (Polymeropoulos et al., 1997; Kam et al., 2018; Pinnell et al., 2021). α-synuclein is a small natively unfolded protein that is predominantly found in soluble form at presynaptic terminals that has been suggested to play a role in homeostasis of the synaptic vesicle pool and the modulation of synaptic transmission *via* sustaining normal SNARE-complex assembly (Kim and Lee, 2008; Burre et al., 2010). During the pathogenesis of PD, it is believed that monomeric α-synuclein assembles into higher ordered structures known as α-synuclein preformed fibrils (PFFs) that contribute death to neuronal cells. Moreover, Increasing experimental evidence suggests that free floating α-synuclein protein seeds and its multiple forms are capable of spreading across different neurons *via* multiple cellular mechanism (Peng et al., 2020; Liu et al., 2022). Recent experimental studies carried out in mice and non-human primates have demonstrated spread of toxic α-synuclein from peripheral organs such as gut and olfactory bulb to central nervous system (Braak et al., 2003; Rey et al., 2016; Kim et al., 2019; Arotcarena et al., 2020). Among multiple cellular pathways reported to mediate release and uptake of α-synuclein, EVs have been demonstrated to play an important role in inter-neuronal transmission of α-synuclein (Danzer et al., 2012; Ngolab et al., 2017; Minakaki et al., 2018). Soluble oligomeric and monomeric species of α-synuclein were found associated with exosomes isolated from neuroblastoma cells, overexpressing the protein (Emmanouilidou et al., 2010). Moreover, exosome-associated α-synuclein were more efficiently taken up by the recipient cells and induced more toxicity compared to free form of α-synuclein (Danzer et al., 2012). Considering the potential role of divalent metal ions in protein aggregation, exposure of cultured dopaminergic neurons with manganese (Mn^2+^) were found to induce secretion of misfolded α-synuclein *via* exosomes. Uptake of these exosomes by the microglial cells elicited neuroinflammatory responses in both cell culture and animal models (Harischandra et al., 2019). Similarly, EVs isolated from CSF of patients with Parkinson’s disease were found to contain a pathogenic species of α-synuclein that could initiate oligomerization of soluble α-synuclein in target cells and confer disease pathology (Danzer et al., 2012; Stuendl et al., 2016). Exosomes containing ganglioside lipids GM1 or GM3 were reported to promote aggregation of α-synuclein (Grey et al., 2015). Recently, it has been found that lipid peroxidation product 4-hydroxynonenal (HNE) increases aggregation of endogenous α-synuclein in primary neurons and trigger secretion of EVs containing cytotoxic oligomeric α-synuclein. Focal administration of these EVs into the striatum of wild-type mice resulted in spread of synuclein pathology to anatomically connected brain regions, thereby confirming direct involvement of EVs in propagating the neurodegenerative process of PD (Zhang S. et al., 2018).

## Extracellular vesicles as biomarkers in neurodegenerative disorders

Presence of misfolded protein aggregates in cerebrospinal fluid (CSF) and blood forms a basis for development of biomarkers of NDs. However, presence of these proteins at extremely low concentration in CSF and blood makes them less attractive for the development of meaningful neurodegenerative-specific biomarkers (Thompson et al., 2016). To overcome these hurdles, it is important to examine other components of the biofluid such as EVs and characterize their association with the progress of the neurological diseases. Secretion of EVs by almost all cell types, presence of cargos such as proteins, lipids, nucleic acid that varies according to their cellular origin makes them promising biomarkers to understand cellular and pathogenic processes of ND (Chiasserini et al., 2014; Vella et al., 2016; Younas et al., 2022). Availability of highly advanced biochemical tools such as high resolution tandem mass spectrometry, bioinformatics, immunological assays and electron microscopy allows comprehensive analysis of EVs isolated from CSF and blood for the presence of specific biomarkers under physiological and pathophysiological conditions.

In several experiments, detection of pathogenic proteins of NDs from CSF-isolated EVs hold promise for the development of early or presymptomatic biomarkers for NDs (Table 2). An analysis of phosphorylated tau (p-tau) isoforms isolated from CSF-derived EVs have identified high levels of p-T181-tau/T181 in AD patients at early stages of disease that decrease at later stages (Saman et al., 2012). Interestingly, the levels of these proteins were significantly higher in patients than in healthy controls, therefore, making them promising biomarkers to study development of AD (Kim et al., 2021). Several other reports have demonstrated presence of other pathogenic proteins (prion/prion-like proteins, α-synuclein) in CSF-derived EVs, making them potential candidates for development of sustained ND therapies (Chiasserini et al., 2014; Stuendl et al., 2016). In addition to misfolded proteins, several studies have investigated the potential of miRNA associated with EVs in detecting or studying the progress of NDs. Dysregulation of miRNA caused due to mutated genes of NDs and protection of miRNA by EVs from degradation makes them an attractive pool as potential biomarkers of NDs. Several studies have reported upregulation of certain microRNAs (miR-9-5p, miR342-3p, and miR-598) in the blood and CSF of AD patients when compare to EVs of healthy individuals (Manna et al., 2020; Wang and Zhang, 2020). In patients with sporadic ALS, downregulation of exosomal miR-1825 and miR-1234-3p that target *NXPH3* and *NLE1* gene involved in pathogenesis of ALS have been seen, suggesting involvement of these genes in pathogenesis of ALS (Taguchi and Wang, 2018; Chen et al., 2021). Taken together, the detection of pathogenic proteins and miRNA from CSF-derived EVs hold great promise to serve as early biomarkers for neurodegenerative diseases. However, the moderately invasive nature of CSF collection, painful procedure to collect CSF from patients and availability of low sample volume limits its widespread use in routine primary clinical care practices (Blennow, 2017).

The ability of central nervous system EVs to cross the blood-brain barrier into blood has opened up a new paradigm for the diagnosis of neurodegenerative diseases (Thompson et al., 2016; Song et al., 2020). CNS-specific protein markers such as CD171 (also known as neural cell adhesion molecule L1, L1CAM) and glutamate aspartate Transporter (GLAST) have been used to isolate brain-derived EVs from blood by immunoprecipitation (Younas et al., 2022). Analysis of AD-related cargos in neuronal EVs (nEVs) revealed presence of gradually increased levels of Aβ-42 along the Alzheimer’s continuum, indicating that nEVs have the potential to reflect brain pathological changes and are emerging as promising liquid biopsy tools. Moreover, they could also serve as a predictor of disease progression during clinical trials. Similarly, another study carried out by Dutta et al. (2021) on brain-derived EVs isolated from serum using neuronal and oligodendroglial markers in two independent cohorts found lower concentration of α-synuclein in control group and higher in Multiple system atrophy (MSA) compared to the Parkinson’s disease (PD) group. Increased levels of tau protein have been detected in nEVs isolated from human plasma, with significantly increased levels found in PD patients compared to AD patients (Shi et al., 2016). Moreover, they found that the ratio of α-synuclein level in oligodendroglial exosomes compared to putative neuronal exosomes could be used as a sensitive marker for distinguishing between PD and MSA (Dutta et al., 2021). Overall, these studies highlighted the therapeutic potential of CNS-derived EVs as biomarkers, evaluating the early detection and progression of NDs. However, due to low-abundance of brain-derived EVs in the blood, development of standardized and ultrasensitive methods to isolate these types of EVs and high resolution visualization techniques are needed to utilize brain-derived EVs as reliable biomarkers for NDs. Moreover, concerns regarding specificity of surface markers used to isolate nEVs and lack of standardized procedure to identify and correlate EVs isolated by different cell types of brain under different conditions needs further investigation (Jia et al., 2019; Li et al., 2019, 2022; Kalluri and LeBleu, 2020; Younas et al., 2022).

## Extracellular vesicles as drug delivery vehicles to central nervous system

Despite significant advancement of nanotechnology, delivery of drugs to a specific tissue is still a major challenge to move different therapies, including the most promising RNA interference (RNAi), from bench to bedside (Park, 2013; Dar et al., 2015a,b). Activation of immune response, inability to cross different biological membranes, biocompatibility and toxicity are some of the major concerns related with the existing drug delivery systems (Keles et al., 2016; Torrice, 2016). Discovery of EVs as intercellular communication vehicles has gained tremendous attention academically as well as at industrial level as reflected by start-up of new international pharmaceutical companies (Evox Therapeutics in United Kingdom, Codiak Bioscience in U.S.A and ExoPharm in Australia), exploring the therapeutic potential of EVs in diagnosis and drug-delivery. These small, nano- sized vesicles play an important role in intercellular communication by delivering payloads in the form of proteins, mRNA and non-coding RNAs (Lakhal and Wood, 2011).

Several intriguing properties of exosomes such as immunological inertness and ability to overcome natural biological barriers have increased their therapeutic potential exponentially in delivering drugs, particularly non-coding RNAs (Luan et al., 2017). The pioneering work carried out by Alvarez-Erviti et al. (2011) to exploit exosomes for delivering siRNA across blood-brain barrier after systemic injection provides the first proof-of-concept for the potential of exosomes as vehicles of drug delivery. In this study, immature dendritic cells of mice were used to isolate EVs. Cells were transfected with plasmid encoding RVG peptide fused to Lamp2b in order to express RVG peptide on the surface of EVs. RVG is a 29-mer peptide isolated from rabies virus glycoprotein that mediates delivery of drugs across the blood-brain barrier. Since then several studies have been carried out to engineer EVs for delivering different kinds of pharmaceutical drugs to the brain (Lakhal and Wood, 2011; Wiklander et al., 2019; Zhou et al., 2022).

Based on their natural tendency to cross biological barriers, biodistribution of unmodified EVs have been carried out after intravenous injections. Majority of the IV injected EVs were found to rapidly accumulate in the reticuloendothelial system, with a small portion reaching to the brain (Lai et al., 2014; Wiklander et al., 2015; Pauwels et al., 2021). Moreover, parental cell surface markers have been found to be important in targeting EVs to a particular tissue. A significantly higher uptake of EVs, purified from neuronal cells, to the brain was observed in mice and zebrafish models (Yang et al., 2015; Venkat et al., 2019). To achieve more targeting of EVs to brain parenchyma, recombinant fusion proteins were expressed on EV surfaces by transfecting cells with the plasmid expressing the desired fusion protein. A well-characterized and highly expressed EV membrane protein such as Lamp2b has been used as an anchor to display brain targeting peptides such as RVG peptide, lactoferrin, transferrin receptor binding T7 peptide and RGDYK peptide on surface of EVs for brain targeting (Alvarez-Erviti et al., 2011; Cooper et al., 2014; Izco et al., 2019; Dar et al., 2021). Using these strategies, a higher accumulation of these EVs were reported in the brain after intravenous injections leading to silencing of genes, causing neurodegenerative diseases (Tian et al., 2018; Kim et al., 2020).

Despite considerable potential of EVs in drug delivery to brain, limited knowledge of EV biogenesis and loading of therapeutic drugs remains one of the major obstacle to harness their full potential as natural drug delivery systems (Lakhal and Wood, 2011; Ramirez et al., 2018; Wiklander et al., 2019). Various techniques like electroporation, direct incubation of hydrophobic drugs with EVs, transfection of cargos into cells for endogenous loading into EVs, have been developed to load therapeutic cargos into EVs (Sun et al., 2010; Alvarez-Erviti et al., 2011; Didiot et al., 2016). Similarly, other methods such as development of anchor peptide against surface markers of EV proteins have been used for loading phosphorodiamidate morpholino oligomer (PMO) onto EVs (Gao et al., 2018). However, these loading methods are limited by low efficiency, toxicity, lack of reproducibility and scalability (Kooijmans et al., 2013). Moreover, these methods produce a heterogenous population of EVs that imposes further complexity in understanding the phenotypic effects of EVs in targeted cells (Sutaria et al., 2017; Murphy et al., 2019). To harness the therapeutic potential of EVs, it is important to understand the intracellular pathways of EV biogenesis, so that the natural characteristics of EVs can be exploited for therapeutic applications rather than by manipulating them in the laboratory. Recently, we developed a new method for loading therapeutic nucleic acid into EVs by utilizing naturally occurring free GAPDH binding sites on the surface of EVs (Dar et al., 2021). Further experiment revealed presence of phosphatidylserine binding domain of GAPDH (designated as G58). Using G58 as an anchoring peptide to EV surface, multiple recombinant fusion proteins were developed that lead to efficient delivery of siRNA into the brain of Huntington’s disease mouse model, resulting in silencing of *HTT* gene in different regions of the mouse brain. Presence of natural free GAPDH-binding sites on EVs isolated from multiple cells is one of the important steps toward developing a universal loading method where EVs from patients could be directly used for loading cargos with minimum *in vitro* manipulations.

## Conclusion

Extracellular vesicles that were initially considered as garbage bags have been shown to play an important role in cell-cell communications. Given their unique characteristics, EVs have attracted immense interest in understating their under physiological and pathophysiological conditions. EVs have been demonstrated to carry pathogenic proteins responsible for spreading of neurodegenerative disease and, therefore, hold great potential for clinical application. Although significant advances have been made in understanding the biology of EV biogenesis and biochemical characteristics, very little is known about the role of different types of EVs in packaging and release of pathogenetic proteins such as prion proteins, Aβ protofilaments and other types of misfolded proteins. Most of the studies carried out in the past have used the term exosomes while describing their roles in NDs without fully characterizing the different types of vesicles obtained from the cultural media and biological fluids. Using the advanced techniques such as single vesicle high-resolution IFC and specific markers for exosomes and microvesicles, the role of different types of EVs could be established (Gorgens et al., 2019; Jeppesen et al., 2019).

Presence of EVs in various biological fluids opened up new possibilities to use EVs as biomarkers for early detection and progression of various pathological states such as neurodegenerative diseases. Recent findings of brain-derived EVs in blood raised a great interest in isolation and biochemical characterization of these EVs as these EVs could offer enormous information to study neurodegeneration in real time. However, heterogeneity of EVs has complicated their molecular characterization. Therefore, future studies need to focus on developing novel methods to isolate different types of EVs and analyze their biochemical signatures. Another emerging application of EVs is their ability to deliver drugs across biological barriers. Biocompatibility of EVs, immunological inertness and ability to express desired protein ligands on their surface using conventional genetic engineering tools have attracted considerable interest in EVs as future delivery systems in gene therapy. However, the EV field is still in infancy and much attention needs to be given to understand how EVs deliver cargos inside cells. Therefore, future studies need to dissect different routes of EV entry into the cells and release of EV cargos into the cytoplasm. Moreover, novel methods for loading drugs to EVs needs to be developed that needs little bioengineering so that patient-derived EVs could be used directly to deliver therapeutic drugs. Taken together, it seems possible that EVs hold tremendous potential to develop as multifunctional biopharmaceuticals.

## Author contributions

GD conceived the study and wrote the review article. ER and RB took part in collecting information about the role of EVs in neurodegenerative disease. ER also helped in writing the manuscript. EN assisted in collecting literature related to the role of EVs in biomarkers and took part in editing the manuscript. AD assisted in editing the manuscript. MB assisted in a survey of literature about the role of EVs in drug delivery. All authors contributed to the article and approved the submitted version.
